# Assessing the genome level diversity of *Listeria monocytogenes* from contaminated ice cream and environmental samples linked to a listeriosis outbreak in the United States

**DOI:** 10.1371/journal.pone.0171389

**Published:** 2017-02-06

**Authors:** Yi Chen, Yan Luo, Phillip Curry, Ruth Timme, David Melka, Matthew Doyle, Mickey Parish, Thomas S. Hammack, Marc W. Allard, Eric W. Brown, Errol A. Strain

**Affiliations:** Center for Food Safety and Applied Nutrition, Food and Drug Administration College Park, MD, United States of America; Institut National de la Recherche Agronomique, FRANCE

## Abstract

A listeriosis outbreak in the United States implicated contaminated ice cream produced by one company, which operated 3 facilities. We performed single nucleotide polymorphism (SNP)-based whole genome sequencing (WGS) analysis on *Listeria monocytogenes* from food, environmental and clinical sources, identifying two clusters and a single branch, belonging to PCR serogroup IIb and genetic lineage I. WGS Cluster I, representing one outbreak strain, contained 82 food and environmental isolates from Facility I and 4 clinical isolates. These isolates differed by up to 29 SNPs, exhibited 9 pulsed-field gel electrophoresis (PFGE) profiles and multilocus sequence typing (MLST) sequence type (ST) 5 of clonal complex 5 (CC5). WGS Cluster II contained 51 food and environmental isolates from Facility II, 4 food isolates from Facility I and 5 clinical isolates. Among them the isolates from Facility II and clinical isolates formed a clade and represented another outbreak strain. Isolates in this clade differed by up to 29 SNPs, exhibited 3 PFGE profiles and ST5. The only isolate collected from Facility III belonged to singleton ST489, which was in a single branch separate from Clusters I and II, and was not associated with the outbreak. WGS analyses clustered together outbreak-associated isolates exhibiting multiple PFGE profiles, while differentiating them from epidemiologically unrelated isolates that exhibited outbreak PFGE profiles. The complete genome of a Cluster I isolate allowed the identification and analyses of putative prophages, revealing that Cluster I isolates differed by the gain or loss of three putative prophages, causing the banding pattern differences among all 3 *Asc*I-PFGE profiles observed in Cluster I isolates. WGS data suggested that certain ice cream varieties and/or production lines might have contamination sources unique to them. The SNP-based analysis was able to distinguish CC5 as a group from non-CC5 isolates and differentiate among CC5 isolates from different outbreaks/incidents.

## Introduction

Listeriosis can have a relatively long incubation period compared to other foodborne illnesses, while the contaminated food products linked to the illness, such as milk, cheese, meat, fresh fruit and vegetable [1], generally have brief shelf lives and may have been discarded before a broad range of samples can be obtained during source tracking [2, 3]. This has consequences for trace-back efforts using whole genome sequencing (WGS), as understanding the diversity among clinical, food and environmental isolates is an important part of identifying which isolates are most likely associated with a given outbreak. However, in 2015 an outbreak of listeriosis in the United States (U.S.) was linked to various ice cream products, which have a much longer shelf life [4], enabling collection of different types of samples manufactured over a relatively long period of time. In addition, *L*. *monocytogenes* was able to survive the freezing storage condition [5]. This, in turn, provided an excellent opportunity to assess the genome-level diversity of *L*. *monocytogenes* that persisted in production facilities. The outbreak investigation started with positive *L*. *monocytogenes* findings in ice cream products made on a production line (Production line A, designated in this article only) of a facility (Facility I, designated in this article only). Subsequently, isolates from 4 elderly patients, who were hospitalized for other medical conditions prior to exposure to the contaminated ice cream, were matched to isolates from ice cream products [6]. In this article, these 4 clinical cases in one hospital are designated as Group I illnesses, with onset dates ranging from January 2014 to January 2015. The identification of contaminated Facility I products led to the subsequent sampling and identification of *L*. *monocytogenes*-positive products in a second facility (Facility II) operated by the same company. A retrospective review of the PulseNet database identified a second cluster of listeriosis cases (serotype 3b) in three states linked to the ice cream products made in Facility II. In this article, these cases are designated as Group II illnesses, with onset dates ranging from January 2010 to October 2014. An environmental sample from a third facility (Facility III) operated by the same company also yielded a *L*. *monocytogenes* isolate; however, no clinical cases were linked to that sample [6].

Prophage variation is also an important source of genomic variability that could serve as markers for epidemiology of *L*. *monocytogenes*. Previous studies have demonstrated that prophages are conserved among isolates associated with the same outbreak [7, 8]; further, such conservation has been observed among isolates that had persisted in a food processing facility for up to 2 years [7]. In contrast, significant prophage diversification, due to recombination, was observed among isolates resident in a food processing facility over the course of 12 years, while only 1 SNP was in the rest of the genome [9]. Prophage variations, such as SNPs in the PFGE restriction sites and gain/loss of prophages, have also been shown to cause *Asc*I-PFGE banding pattern changes among genetically close isolates [8, 10, 11]. Due to the availability of a complete genome for one of the isolates from this ice cream-associated outbreak, we were able to investigate prophage variations.

*L*. *monocytogenes* consists of four evolutionary lineages [1, 12]. Within each lineage, the population is further structured into clonal groups, the identification of which is very useful for understanding the biodiversity of *L*. *monocytogenes* related to pathogenicity and epidemiology. Isolates within each clonal group are relatively closely related and isolates between different clonal groups are relatively distant from each other, as illustrated by two core genome MLST schemes [13, 14]. A system to define clonal groups is clonal complex (CC)/singleton, defined by a 7-gene MLST scheme [15]. The sequence types (STs) in the same CC differ by not more than one allele from at least one other ST. A singleton exhibits an ST that differs from all other existing STs by at least two alleles, meaning a singleton does not belong to any existing CCs [15]. Our preliminary analysis showed that isolates associated with this ice cream outbreak had MLST ST5, which belongs to clonal complex 5 (CC5), a clonal group of serotypes 1/2b and 3b contaminating a variety of food products and processing environments and/or causing outbreaks [2, 16–19]. A clonal complex that had been involved in at least two outbreaks is also classified as an epidemic clone and CC5 was one of the most diverse epidemic clones [13, 14]. Although reference-based SNP methods have been shown to perform well for closely related isolates from a single outbreak [20, 21], further validation is necessary to determine their usefulness for inferring the phylogeny of slightly more diverse isolates in the same clone but from different outbreaks. Mapping quality and subsequent SNP calling by reference-based WGS methods, may be affected by ascertainment bias when these methods were applied to a set of relatively diverse isolates [22].

The objectives of the present study were to 1) describe the SNP-based WGS analyses on the food, environmental and clinical isolates, most of which were analyzed during the outbreak investigation, 2) investigate the prophage variations among one cluster of outbreak-associated isolates, for which a complete genome was available and 3) determine whether SNP-based analysis can be used for simultaneous identification of CC5 and differentiation of CC5 strains from different outbreaks/incidents.

## Materials and methods

### *In silico* serotyping, WGS and PFGE

The WGS and PFGE data for all isolates were obtained from GenomeTrakr and PulseNet. Two set of genomes were collected. The first set of genomes was from food, environmental and clinical isolates sequenced during the initial outbreak investigation. These isolates were collected using enrichment-based isolation according to the *L*. *monocytogenes* chapter of FDA *Bacteriological Analytical Manual* [23]. The second set of genomes was from isolates collected from ice cream scoops using two methods, 7 by enrichment-based Most Probable Number (MPN) enumeration and 29 by enrichment-free direct plating enumeration in a *L*. *monocytogenes* enumeration study for ice cream scoops [5, 24] and a growth kinetics study for milkshakes [25]. These isolates were not subject to PFGE analysis. *In silico* serotyping was performed using tools built in the BIGSdb-Lm database (http://bigsdb.pasteur.fr/listeria/) [13]. WGS analyses grouped all the isolates into two clusters and a branch containing a single isolate (S1 Fig). Cluster I contained 4 clinical isolates, 78 food isolates representing different ice cream varieties and lots manufactured between August 2014 and March 2015 on Production line A of Facility I, and 4 environmental isolates collected from Facility I during the outbreak investigation (S1 Table). Cluster II contained 5 clinical isolates, 21 food isolates representing ice cream varieties and lots manufactured between April 2014 and March 2015 in Facility II, and 30 environmental isolates from Facility II collected during the outbreak investigation, as well as 4 isolates from ice cream manufactured between March and April 2015 on Production line B of Facility I (S1 Table). Among the 30 environmental isolates from Facility II, 28 were obtained from 14 samples (2 isolates per sample). The single branch contained one isolate, from the only sample in Facility III, and thus no further analyses were performed on this isolate.

### SNP-based WGS analysis

The SNPs among all isolates were identified using the default settings of the Center for Food Safety and Applied Nutrition (CFSAN) SNP Pipeline version 0.6.1 [20, 26] that targets the entire genome, including coding and non-coding regions from the core and accessory genomes. Briefly, WGS raw reads from each outbreak-associated isolate were mapped to the reference genomes using Bowtie2 version 2.2.2 [27]. The resulting BAM file was sorted using Samtools version 1.3.1 [28], and a pileup file was generated for each sample. These files were then processed using VarScan2 version 2.3.9 to identify high quality variant sites using the mpileup2snp option [29]. The Python script was then used to parse the.vcf files and construct a SNP matrix. GARLI was used to infer topologies based on that SNP matrix [30]. We first chose an ice cream isolate (CFSAN029793), for which we had a fully closed genome (GenBank Accession NZ_CP016213.1, https://www.ncbi.nlm.nih.gov/nuccore/NZ_CP016213.1), as the reference to perform phylogenetic analysis on all isolates. Because accuracy of SNP calling by reference-based methods may be reduced when these methods were applied to slightly more diverse isolates [22, 31], we then performed separate phylogenetic analyses on individual clusters identified in the initial analysis, by using CFSAN029793 as the reference for Cluster I and a draft genome of an ice cream isolate (CFSAN030683, GenBank Accession NZ_MAGN00000000.1, https://www.ncbi.nlm.nih.gov/nuccore/NZ_MAGN00000000.1) as the reference for Cluster II. When analyzing isolates of each cluster, we also identified non-outbreak isolates from GenomeTrakr and PulseNet that matched the outbreak isolates by PFGE/MLST, performed preliminary WGS analyses, and then selected genomes that were most closely related to the outbreak-associated isolates for comparison. The three PFGE/MLST-matched, non-outbreak isolates for Cluster I were CFSAN020389 (PFGE profile P1), CFSAN029618 (PFGE profile P5) and CFSAN022649 (PFGE profile P4). We could not found non-outbreak isolates that matched outbreak-associated isolates in Cluster II by the two-enzyme PFGE; therefore, we chose two non-outbreak isolates, CFSAN021784 and PNUSAL000243, which exhibited the MLST ST in all Cluster II isolates and the *Asc*I-PFGE profile observed in most of the outbreak isolates in Cluster II (S1 Table). For each cluster, two CFSAN SNP Pipeline analyses were performed. The initial WGS analysis included isolates in each cluster and isolates epidemiologically unrelated to the cluster to show that WGS distinguished outbreak isolates from PFGE/MLST-matched, but epidemiologically unrelated isolates. The second analysis included only isolates in each cluster. For each cluster of closely related isolates, the SNP Pipeline applied a filter to exclude variant sites in high density variant regions (≥3 variant sites in ≤1000 bp of any one genome) since they may be the result of recombination, low quality sequencing/mapping and/or be associated with repetitive elements. Four regions, 55 bp (containing 9 variant sites), 34 bp (5 sites), 28 bp (10 sites), and 84 bp (8 sites) were excluded for Cluster I; and four regions, 3 bp (3 sites), 37 bp (3 sites), 27 bp (6 sites) and 260 bp (10 sites), were excluded for Cluster II. In addition, 16 variant sites within 500 bp of either end of any reference genome contigs were excluded by the Pipeline for Cluster II because in general less reads were mapped to the end of contigs, resulting in lower quality of mapping and assembly.

### Prophage analysis of Cluster I isolates

A combination of PHAST [32] and PHASTER [33] was used to identify putative prophages in the complete genome of CFSAN029793. PHAST was used to identify insertion sites and PHASTER was used to identify the start and end of each prophage. Then the presence/absence of these putative prophages in draft genomes was determined by a combination of two approaches: 1) the insertion sites of the CFSAN029793 prophages were identified in the CLC Genomics WorkBench 8.5.1 (Aarhus, Denmark)-assembled draft genomes by BLAST and the sequences adjacent to the insertion sites were compared to the prophages of CFSAN029793, and 2) the CFSAN029793 prophages were searched against draft genomes by BLAST and a threshold of ≥ 60% query coverage with ≥80% sequence identity [34, 35] of BLAST alignment indicated the presence of a CFSAN029793 prophage in a draft genome.

### Clonal complex 5 analysis

*In silico* MLST was performed using CLC Genomics WorkBench 8.5.1 (Aarhus, Denmark) and the MLST profiles defined in the PasteurMLST *L*. *monocytogenes* database (now in BIGSdb-Lm, http://bigsdb.pasteur.fr/listeria). The CFSAN SNP Pipeline was used to construct a phylogeny of a subset of isolates taken from this ice cream outbreak as well as isolates from previous CC5-associated outbreaks/incidents: 2011 U.S. cantaloupe outbreak [17], 2013 U.S. Hispanic-style cheese outbreak [16], and serotype 1/2b isolates from the 2014 U.S. stone fruit recall [36]. We chose isolates exhibiting different PFGE profiles observed in those incidents. To avoid using only ST5 to represent CC5, we included a CC5 isolate (CFSAN028312) of ST745 in the analysis. Four non-CC5 serotype 1/2b isolates were chosen for comparison: the environmental isolate from Facility III, CFSAN032502 (singleton ST489, the environmental isolate from Facility III), F4233 (ST3 of CC3), LM07-01067 (ST386 of CC224), CFSAN003423 (ST379 of CC379) and CFSAN003438 (ST379 of CC379); these STs differed from ST5 by 4, 5, 6, 2 and 2 MLST alleles, respectively.

## Results

### All isolates

All clinical isolates and all food and environmental isolates from the three facilities belonged to the molecular serogroup IIb, which is comprised of serotypes 1/2b, 3b and 7, and belonged to lineage I [37]. Two WGS clusters and a branch containing a single isolate were identified (S1 Fig). Cluster I consisted of food isolates from Production line A of Facility I, environmental isolates from Facility I, and clinical isolates, exhibiting MLST ST5. Cluster II consisted of food isolates from Production line B of Facility I, food and environmental isolates from Facility II, and clinical isolates, exhibiting MLST ST5. This suggested that different production lines of Facility I might have their unique contamination sources. The single WGS branch contained an environmental isolate from Facility III and belonged to singleton ST489. No clinical cases were linked to this isolate.

### Cluster I PFGE and WGS analysis

Cluster I was comprised of 82 food and environmental isolates from Facility I and 4 clinical isolates from 4 patients (Group I illnesses). A total of 9 two-enzyme (*Asc*I and *Apa*I) PFGE profiles, with brief designations of P1 through P9, were observed from all food and environmental isolates (S1 Table). Three of these profiles matched the 3 PFGE profiles (P1, P5 and P6) of the 4 clinical isolates (Group I illnesses). A total of 3 *Asc*I-PFGE profiles were observed in all the clinical isolates, as well as in all the food and environmental isolates. The outbreak-associated isolates, albeit exhibiting 9 PFGE profiles, were clustered together by WGS; in contrast, non-outbreak isolates (CFSAN020389, CFSAN029618 and CFSAN022649) that exhibited the outbreak PFGE profiles were placed outside the outbreak cluster (S2 Fig). These unrelated isolates differed from outbreak-associated isolates by at least 71 SNPs. This clustering supports the epidemiological findings that ice cream products manufactured on Production line A were the likely cause of the Group I illnesses. Our reference-based approach was able to identify the SNPs that specifically distinguished the entire outbreak cluster from the unrelated isolates, which could be used for future functional genomics (S2 Table).

**Fig 1:**
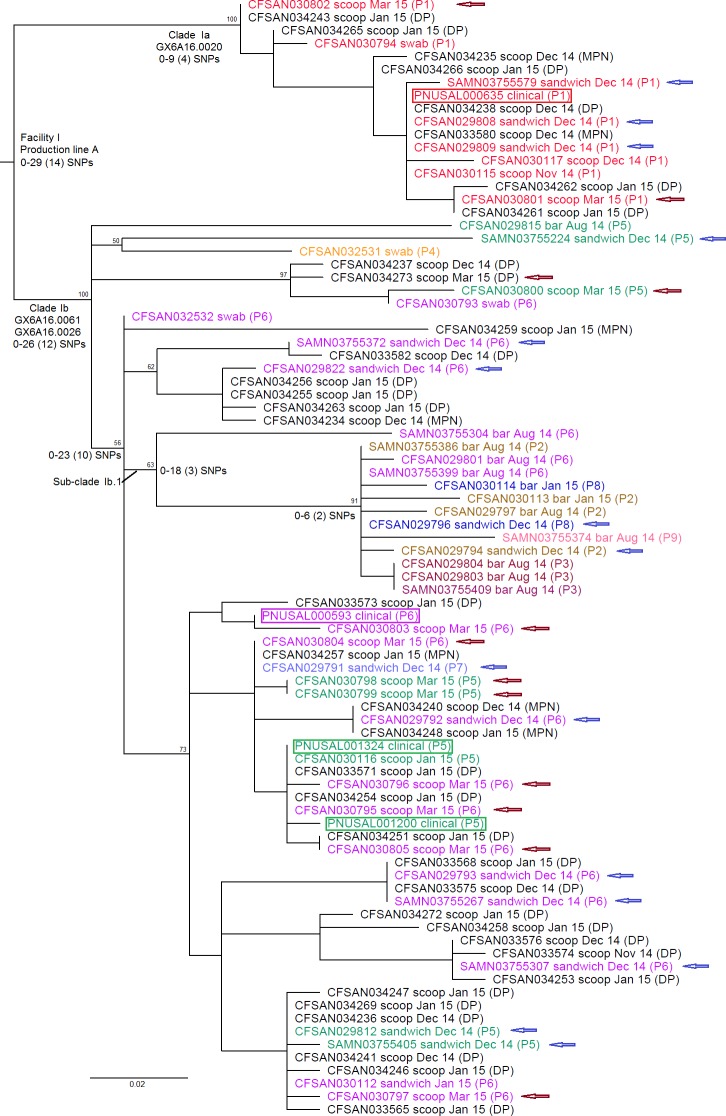
Maximum likelihood tree of Cluster I isolates. Isolates were obtained from Group I illnesses, food samples produced on Production line A of Facility I, and environmental samples from Facility I. The SNP matrix was generated using CFSAN029793 as the reference and contained 152 polymorphic loci. The tree uses midpoint rooting. Isolate identifier is followed by sample type, abbreviation of production date of the food samples, and available PFGE profile in the parenthesis. Isolates of the same PFGE profile are in the same color and isolates without PFGE information are in black. The 36 isolates without PFGE profiles were obtained from *L*. *monocytogenes* enumeration and growth kinetics studies [5, 25] and the method of isolation (direct plating (DP) or most probable number (MPN)) is listed in the parenthesis after each isolate ID. The 4 clinical isolates are highlighted in boxes. Blue arrows denote isolates from sandwiches produced in December 2014. Brown arrows denote isolates from scoops produced in March 2015. The bootstrap values for major clades are listed on top of the root of each clade. The *Asc*I-PFGE profiles, the minimum and maximum numbers of SNPs with the medians in the parenthesis are listed near the root of each clade.)

To precisely determine the SNP differences among all the outbreak isolates, we then performed a second WGS analysis on only the outbreak-associated isolates, identifying a SNP matrix of 152 polymorphic loci. The 86 isolates differed by 0 to 29 (median, 14) SNPs, calculated without counting gaps (Fig 1). The 4 clinical isolates differed by 1 to 19 (median, 10.5) SNPs. Out of the 50 isolates with PFGE information, 38 that exhibited clinical PFGE profiles (P1, P5 and P6) differed by 0 to 27 (median, 15) SNPs. Any food or environmental isolate differed from their most genetically close clinical isolate by 0 to 16 SNPs, indicating these food and environmental isolates were associated with the clinical cases. Two major clades were identified within Cluster I, Clade Ia isolates exhibiting only PFGE profile P1 (*Asc*I-PFGE profile, GX6A16.0020) and Clade Ib isolates exhibiting the other 8 PFGE profiles (*Asc*I-PFGE profiles, GX6A16.0061 and GX6A16.0026) (Fig 1). Clade Ib isolates differed by 0 to 26 (median, 12) SNPs, and 29 of them that exhibited clinical PFGE profiles (P5 and P6) differed by 0 to 25 (median, 11) SNPs. The sub-clades in Clade Ib were not associated with specific PFGE profiles or food production dates. For example, isolates collected from ice cream sandwiches, taken from a lot produced in December 2014, exhibited the PFGE profiles of P1, P2, P5, P6, P7 and P8, and were distributed throughout Cluster I (noted by blue arrows in Fig 1). Isolates collected from ice cream scoops, taken from two lots produced on two consecutive days in March 2015, exhibited the PFGE profiles of P1, P5 and P6, and were also distributed throughout Cluster I (noted by brown arrows in Fig 1). Isolates collected from other ice cream scoops exhibited the PFGE profiles of P1 and P5, and were again distributed throughout Cluster I (scoops that are not noted by any arrow in Fig 1). Nine out of 10 isolates from ice cream produced in August 2014 were in Sub-clade Ib.1, but 4 other isolates in this sub-clade were from ice cream produced in December 2014 and January 2014. Eleven out of the 12 isolates from ice cream bars are in Sub-clade Ib.1 and the other 2 isolates in this sub-clade were from sandwiches. This led us to believe that this sub-clade may be strongly associated with the ice cream bars, and thus, it is likely that there was a contamination source unique to these ice cream bars. The isolates from enrichment-free direct plating enumeration and enrichment-based detection and enumeration methods [5, 25] were distributed throughout Cluster I, and did not form distinct clades within Cluster I; indicating it is unlikely that any bias was introduced by the enrichment process.

### Cluster I prophage analysis

PHASTER identified four putative prophages from the complete genome of CFSAN029793 (Table 1). Putative prophage 1 was an intact prophage inserted downstream of tRNA-Arg TCT (non-disrupting). Putative prophage 2 was an intact prophage inserted into *comK* (disrupting). PHAST/PHASTER first identified prophage 2 as between position 1856471 and 1896917. The examination of *comK* insertion site subsequently modified the position of this prophage to be between 1856189 and 1896515. Putative prophage 3 was an intact prophage inserted upstream of tRNA-Arg CCG (non-disrupting). Putative prophage 4 was an incomplete prophage with no insertion sites identified. For outbreak-associated isolates, the BLAST alignment of any CFSAN029793 prophage against a draft genome always yielded >90% query coverage with >99% sequence identity when the examination of insertion sites confirmed insertion of that prophage in the draft genome (Table 2). Not all BLAST alignment had 100% coverage probably because draft sequencing randomly missed certain prophage loci. The BLAST alignment of any CFSAN029793 prophage to a draft genome always yielded <40% coverage when the examination of insertion sites revealed no insertion of that prophage in the draft genome (Table 2). The coverage was not 0 because some coding sequences from different prophages could be homologous, due to the mosaic nature of prophages. Cluster I isolates differed in the presence/absence of prophages 1, 2 and 3 and several prophage presence/absence profiles were identified among all isolates; putative prophage 4 was present and conserved in all isolates. Outbreak-associated isolates exhibiting the same *Asc*I-PFGE profile always had the same prophage presence/absence profile. In addition, the same prophage present in different outbreak-associated isolates was conserved, containing 2 or fewer SNPs (Table 2).

**Table 1:** Putative prophages of the complete genome CFSAN029793 identified by a combination of PHAST [32], PHASTER [33] and prophage insertion site examination.

**Prophage**	**Length (kbp)**	**Completeness**	**Insertion site**	**Position**^**a**^	**Possible phage match and NCBI Accession**
1	55.0	Intact	tRNA-Arg TCT	693457–748456	*Listeria* LP-101 (NC_024387)
2	40.3^b^	Intact	*comK*	1856189-1896515^b^	*Listeria* A118 (NC_003216)
3	41.8	Intact	tRNA-Arg CCG	2033993–2075835	*Listeria* A006 (NC_009815)
4	10.7	Questionable		2604325–2615053	*Listeria* A118 (NC_003216)

^a^Position based on the complete genome of CFSAN029793. PHAST was used to identify prophage insertion sites and PHASTER was used to identify the start and end of prophages.

^b^The entire region was first identified by PHAST/PHASTER. The subsequently identified insertion site, *comK*, was examined to slightly modify the PHASTER-identified start and end positions of this prophage.

**Table 2:** Presence/absence and SNPs of CFSAN029793 prophages in Cluster I and PFGE-matched, non-outbreak isolates.

Isolate identifiers and PFGE profiles	Prophage variations			
CFSAN029793^a^ (P6, GX6A16.0061/ GX6A12.0026^b^)	Phage 1	Phage 2	Phage 3	Phage 4
CFSAN030117 (P1, GX6A16.0020/ GX6A12.0227)	+ (0) ^c^	-^d^	-	+ (0)
CFSAN020389 unrelated to the outbreak (P1)	+ (80%) ^e^	-	-	+ (1)
CFSAN030113 (P2, GX6A16.0026/ GX6A12.0489)	+ (1)	+ (1)	-	+ (0)
CFSAN029803 (P3, GX6A16.0026/ GX6A12.0077)	-	+ (1)	-	+ (0)
CFSAN032531 (P4, GX6A16.0026/ GX6A12.0094)	+ (0)	+ (1)	-	+ (0)
CFSAN022649 unrelated to the outbreak (P4)	-	+ (30%)^f^	-^f^	+ (1)
SAMN03755224 (P5, GX6A16.0026/ GX6A12.0227)	+ (0)	+ (2)	-	+ (0)
CFSAN029618 unrelated to the outbreak (P5)	+ (75%)	+ (76%)	-	+ (2)
CFSAN029822 (P6, GX6A16.0061/ GX6A12.0026)	+ (0)	+ (2)	+ (0)	+ (0)
CFSAN029791 (P7, GX6A16.0061/ GX6A12.1512)	-	+ (1)	+ (0)	+ (0)
CFSAN030114 (P8, GX6A16.0061/ GX6A12.2551)	+ (0)	+ (1)	+ (0)	+ (0)
SAMN03755374 (P9, GX6A16.0061/ GX6A12.2358)	-	+ (1)	+ (0)	+ (0)

^a^The criteria to select the isolates for inclusion in the table: each PFGE profile is represented by one isolate. If prophage in multiple isolates exhibiting that PFGE profile differed from the corresponding CFSAN029793 prophage, an isolate with the largest number of SNPs (2 for any prophage) is listed.

^b^Brief and full two-enzyme PFGE designations of each isolate are in the parenthesis.

^c^+(integer), the presence of each prophage was confirmed by the examination of prophage insertion sites, and its BLAST alignment with the corresponding CFSAN029793 prophage yielded ≥90% query coverage with ≥99% sequence identity. The number of SNP differences is in parenthesis.

^d^-, the absence of each prophage was confirmed by the examination of prophage insertion sites and <60% coverage of its BLAST alignment with the corresponding CFSAN029793 prophage.

^e^+(percentage), the presence of each prophage was confirmed by the examination of insertion sites, and its BLAST alignment with the corresponding CFSAN029793 prophage yielded 75% to 80% coverage with 89–95% identity with the exception listed in footnote f. The BLAST coverage is in parenthesis.

^f^CFSAN022649 had a prophage inserted in the insertion site (*comK*) of CFSAN029793 prophage 2, but it aligned to CFSAN029793 prophage 2 for only 30% coverage. That prophage aligned to CFSAN029793 prophage 3 for 68% coverage, but it was inserted into *comK*, not near the tRNA.

Prophage variations were more discriminatory than PFGE for differentiating unrelated isolates because there was greater prophage divergence between outbreak-associated isolates and non-outbreak isolates that were matched by PFGE, using CFSAN029793 as the reference for comparison. When the examination of insertion sites confirmed a prophage insertion in a non-outbreak isolate, its BLAST alignment with CFSAN029793 prophages only yielded 75–80% coverage with 89–95% sequence identity. For example, PFGE-matched CFSAN30117 and CFSAN020389 both possessed CFSAN029793 prophage 1; however, CFSAN029793 prophage 1 aligned with the outbreak isolate (CFSAN30117) for 100% coverage while aligned with the non-outbreak isolate (CFSAN020389) for 80% coverage. Similar results were observed when CFSAN029793 prophages 1 and 2 aligned to PFGE-matched outbreak isolate, SAMN03755224, and non-outbreak isolate, CFSAN029618 (Table 2). The prophage variation between PFGE-matched outbreak isolate, CFSAN032531, and non-outbreak isolate, CFSAN022649, was more complicated. The prophage variation between CFSAN032531 and reference CFSAN029793 only involved the loss of prophage 3. In contrast, the prophage variation between CFSAN022649 and CFSAN029793 appeared to involve the loss of prophages 1 and partial replacement of prophage 2 by prophage 3. Specifically, CFSAN022649 had a prophage inserted in the insertion site (*comK*) of CFSAN029793 prophage 2, but that prophage aligned with CFSAN029793 prophage 2 for only 31% coverage. Instead, this CFSAN022649 *comK* prophage aligned with CFSAN029793 prophage 3 for 68% coverage, while the genomic region of CFSAN022649 next to the insertion site (tRNA-arg) of CFSAN029793 prophage 3 aligned with CFSAN029793 prophage 3 for only 2% coverage.

**Fig 2:**
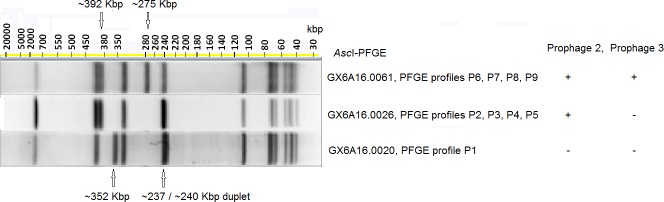
*Asc*I-PFGE banding pattern changes due to the gain/loss of prophages 2 and 3. The *Asc*I-PFGE profile and corresponding brief two-enzyme PFGE profiles are listed on the right of the gel images. The corresponding prophage gain/loss profiles are listed to the right. + indicates the gain of a prophage and–indicates the loss of a prophage. Isolates exhibiting GX6A16.0061 contained both prophages 2 and 3. The loss of prophage 3 resulted in the change of a ~275 Kbp fragment in the gel pattern of GX6A16.0061 to ~237 Kbp in the gel patterns of GX6A16.0026 and GX6A16.0020, and this ~237 Kbp fragment and a ~240 Kbp fragment formed a duplet. The loss of prophage 2 resulted in the change of a ~392 Kbp fragment in the gel patterns of GX6A16.0061 and GX6A16.0026 to ~352 Kbp in the gel pattern of GX6A16.0020. The loss of both prophages 2 and 3 resulted in the pattern change from GX6A16.0061 to GX6A16.0020.

None of the SNPs identified among Cluster I isolates were in the *Asc*I or *Apa*I restriction sites of CFSAN029793. The gain/loss of prophages caused the *Asc*I-PFGE banding pattern differences as discussed below (Fig 2). BLAST analyses showed that the loss of prophage 1, 2 and 3 corresponded to the deletion of ~43 Kbp, ~ 40 Kbp and ~38 Kbp DNA fragment, respectively (Table 1). Prophage 1 was in a ~887 Kbp DNA restriction fragment (between *Asc*I restriction sites at positions 662320 and 1549005 of the reference genome). The loss of 43Kbp from this large fragment was not resolved by PFGE and thus did not contribute to the gel banding pattern changes. Therefore, the *Asc*I-PFGE banding pattern changes were caused by the gain/loss of prophages 2 and 3 (Fig 2). Isolates exhibiting *Asc*I-PFGE profile of GX6A16.0061 (observed in PFGE profiles P6, P7, P8 and P9) contained prophages 2 and 3. The ~38 Kbp deletion resulted from prophage 3 loss caused the shift of a ~275 Kbp DNA fragment (between *Asc*I restriction sites at positions 1941282 and 2216420 of the reference genome) to ~237 Kbp, and this ~237 Kbp fragment and a ~240 Kbp fragment (between *Asc*I restriction sites at positions 373927 to 613583) formed a duplet, which explained the *Asc*I-PFGE gel pattern change from GX6A16.0061 to GX6A16.0026 (observed in PFGE profiles P2, P3, P4 and P5). The ~ 40 Kbp deletion resulted from prophage 2 loss caused the shift of a ~392 Kbp DNA fragment (between *Asc*I restriction sites at positions 1549005 and 1941282 of the reference genome) to ~352 Kbp, which explained the *Asc*I-PFGE gel pattern change from GX6A16.0026 to GX6A16.0020 (observed in PFGE profile P1). The loss of both prophage 2 and 3 resulted in the pattern change from GX6A16.0061 to GX6A16.0020.

### Cluster II PFGE and WGS analysis

**Fig 3:**
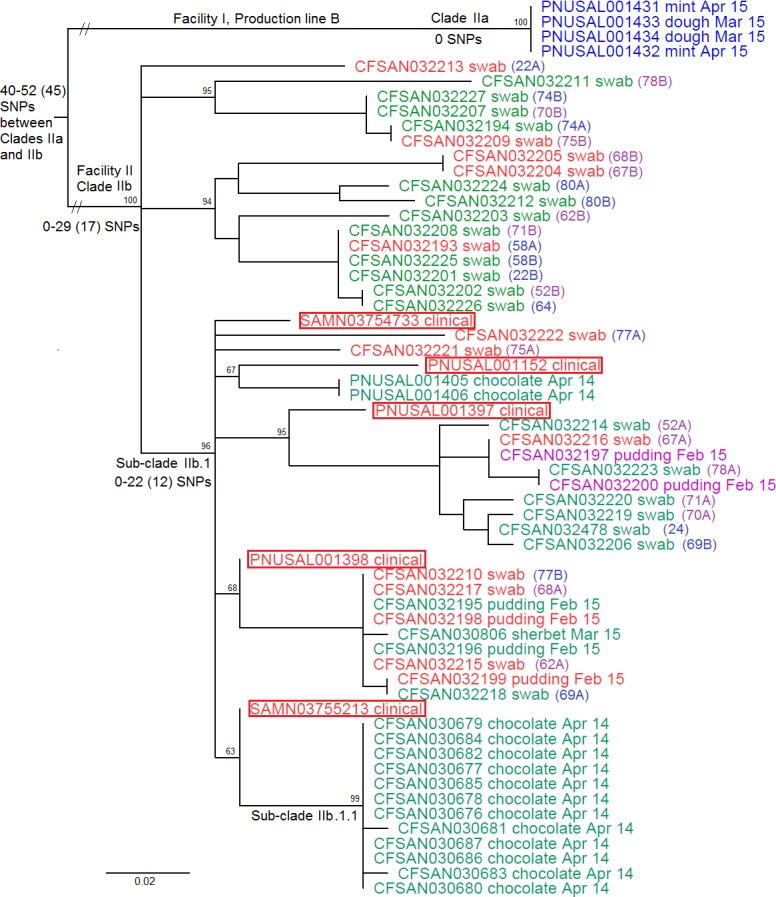
Maximum likelihood tree of Cluster II isolates. Isolates were collected from ice cream produced in Production line B of Facility I (Clade IIa), ice cream/environment from Facility II and Group II illnesses (Clade IIb). The SNP matrix was generated using CFSAN030683 as the reference and contained 169 polymorphic loci. The tree uses midpoint rooting. Isolate identifier is followed by sample type and abbreviation of collection date. Isolates from environmental samples are followed by the sample ID in the parenthesis. Environmental sample IDs ending with A and B indicate two colonies from the same sample. Purple color for an environmental sample ID indicates that for that sample one colony is inside Sub-clade IIb.1 and the other colony is outside Sub-clade IIb.1. Blue color for an environmental sample ID indicates that for that sample both colonies are either inside or outside Sub-clade IIb.1. Only one colony was picked from each of the two environmental samples (024 and 64) and their sample IDs are in blue. Isolates exhibiting PFGE profiles P10, P11, P12 and P13 are printed in blue, red, green and purple, respectively. The clinical isolates, all placed inside Sub-clade IIb.1, are highlighted in boxes. The bootstrap values of major clades are listed on top of the root of each clade. The minimum and maximum numbers of SNPs with median in the parenthesis are listed near the root of major clades.

Cluster II was comprised of 51 food and environmental isolates from Facility II, 4 food isolates from the Production line B of Facility I, and 5 clinical isolates from the 5 patients (Group II illnesses). These isolates exhibited 4 different PFGE profiles (P10, P11, P12 and P13) and all 5 clinical isolates exhibited P11 (S1 Table). The epidemiologically unrelated ST5 isolates (CFSAN021784 and PNUSAL000243) were clearly placed outside Cluster II (S3 Fig); these isolates differed from Cluster II isolates by at least 142 SNPs. In order to precisely determine SNP differences, we then removed the epidemiologically unrelated isolates and performed a second WGS on only the Cluster II isolates, identifying a SNP matrix of 169 polymorphic loci. The 4 isolates from Facility I (PFGE profile P10) formed a clade (Clade IIa), and the 51 isolates from Facility II and 5 clinical isolates formed another clade (Clade IIb) (Fig 3). This clustering, along with PFGE, supports the epidemiologic finding that ice cream produced in Facility II were the likely cause of the Group II illnesses, and that ice cream produced in Facility I may not be linked to Group II illnesses. However, Clade IIa and Clade IIb isolates differed by 40 to 52 SNPs, indicating a relatively close relationship; thus, it is possible that isolates from the two clades might have a common ancestor outside Facility II. We subsequently identified the SNPs that specifically distinguished between Clade IIa and Clade IIb isolates (S2 Table).

Clade IIb isolates differed from each other by 0 to 29 (median, 17) SNPs. The five clinical isolates differed by 2 to 14 (median, 8) SNPs. The isolates exhibiting the clinical PFGE profile (P11) differed by 0 to 26 (median, 15) SNPs. Any food and environmental isolates in Clade IIb differed from its most genetically close clinical isolate by 5 to 16 (median, 11) SNPs, indicating these food and environmental isolates were associated with the clinical cases. Isolates exhibiting PFGE profiles P11, P12 and P13 did not form distinct sub-clades within Clade IIb (Fig 3). The Sub-clade IIb.1 isolates that exhibited PFGE profile P11 differed by 0 to 20 (median, 11) SNPs. Food isolates were placed only in Sub-clade IIb.1, and isolates placed outside Clade IIb.1 were only from environmental samples. Twenty-eight isolates were obtained from 14 environmental samples with two isolates from each sample. For each of the samples 22, 74, 80 and 58, both isolates were outside Clade IIb.1. For each of the samples 69 and 77, both isolates were inside Clade IIb.1. In contrast, for each of the samples 78, 71, 70, 75, 62, 67, 68, and 52, one isolate was placed inside Clade IIb.1 and the other was outside Clade IIb.1. Thus, it is also possible that the facility locations where samples 22, 74, 80 and 58 were collected had not been directly cross-contaminated with ice cream products, although only 2 isolates analyzed for each sample were not sufficient to make definitive conclusions. Within Sub-clade IIb.1, Sub-clade IIb.1.1 contained 12 of all the 14 isolates collected from chocolate ice cream and no isolates from other ice cream products, indicating that there might be a contamination source unique to chocolate ice cream.

### Clonal complex analysis

**Fig 4:**
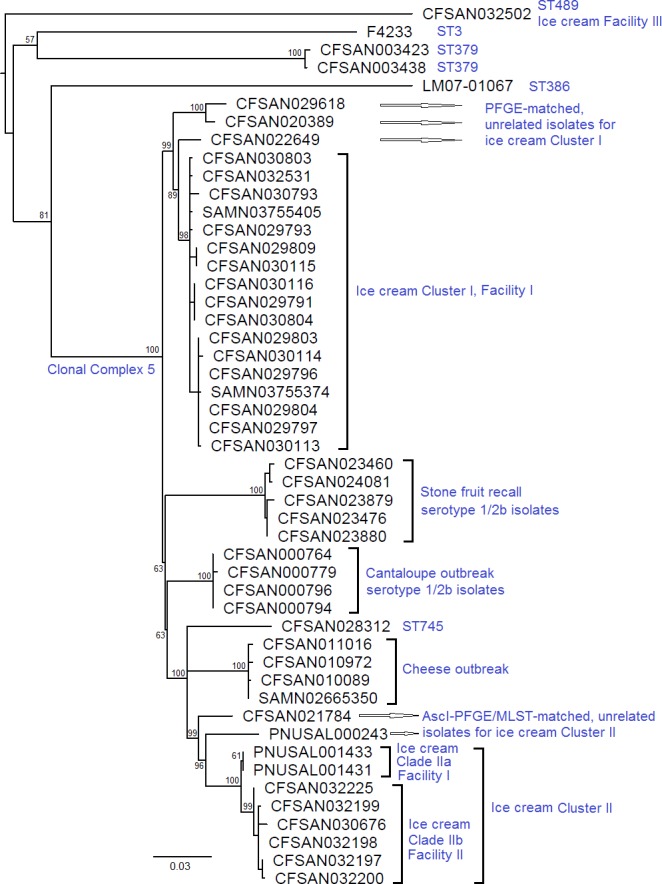
Phylogenetic analysis of Clonal complex (CC) 5 strains from several outbreaks/incidents and other serogroup IIb isolates. The tree uses midpoint rooting. Bootstrap values of major clades are listed near the root of each clade. Serogroup IIb strains differing from CC5 strains by two or more multilocus sequence typing (MLST) alleles were used for comparison. CC5 isolates formed a cluster, within which isolates from each of the cantaloupe, cheese, stone fruit, and ice cream outbreak/incident were all sequence type (ST) 5 and formed distinct clades. The PFGE-matched, epidemiologically unrelated isolates for Clusters I and II of the ice cream outbreak were distinguished from Cluster I and II isolates. The non-ST5 CC5 strain (CFSAN028312, ST745) is placed outside clades representing all other incidents. Within the CC5 cluster, ST5 isolates do not form a distinct clade to be separated from the ST745 strain.

The SNP-based WGS analysis clearly clustered together ST5 isolates from different outbreaks/incidents and a non-ST5 CC5 isolate (CFSAN028312, ST745), when compared to non-CC5 serotype 1/2b isolates (Fig 4). Within the CC5 cluster, our SNP-based analysis was also able to differentiate isolates from epidemiologically unrelated outbreaks/incidents, which PFGE failed to achieve (S4 Fig). ST5 isolates did not form a distinct clade separate from ST745, thus in this case WGS clustering was consistent with the CC but not ST. The PFGE-matched, epidemiologically unrelated isolates for Clusters I and II of the ice cream outbreak were distinguished from Cluster I and II isolates, and they were more close to the ice cream Cluster I and II isolates than to isolates from other incidents.

## Discussion

The initial recognition of this outbreak, as well as the 2014 stone fruit outbreak [36, 38], started with pathogen finding in food products and DNA fingerprinting of food isolates, followed by the identification of clinical cases involved in the outbreaks [6]. This highlights the value of real-time WGS-based surveillance of *L*. *monocytogenes* from food and environmental sources even when they are not yet implicated by any epidemiological evidence. U.S. federal and state agencies initiated a real-time *Listeria* project, in which Food and Drug Administration (FDA) and Department of Agriculture primarily sequence food and environmental isolates and Centers for Disease Control and Prevention (CDC) primarily sequences clinical isolates. Fully sequenced genomes are stored at National Center for Biotechnology Information (NCBI) [39, 40], which generates a daily-updated single nucleotide polymorphism (SNP)-based whole genome sequencing (WGS) tree (https://www.ncbi.nlm.nih.gov/pathogens/isolates/). A signal of clustering (e.g., a WGS match between food/environmental and clinical isolates) could serve as an early warning, to be followed up by epidemiological investigation and additional WGS analyses, e.g. reference-based SNP analysis [41] and whole genome multilocus sequence typing (MLST) analysis [40].

In this study, we analyzed WGS of isolates from multiple product varieties and lots manufactured in two facilities that were linked to the clinical cases. Isolates from ice cream bars in Facility I (in WGS Cluster I) and isolates from chocolate ice cream in Facility II (in WGS Cluster II) appeared to have common sources unique to each of them. However, neither raw material was collected nor was sufficient environmental sampling performed to precisely determine these sources. The production lines A and B of Facility I might have different sources of contamination. Certain environmental samples from Facility II may not have direct cross contamination with the products. Other than these, we did not find strong association between WGS clades and product varieties, production dates. The relatively long shelf life of ice cream allowed collection of samples yielding isolates that contributed to the overall genomic diversity. For example, PFGE profiles P3 and P9 were only observed in isolates from ice cream bars produced in August 2014 and collected in February 2015. The collection of these ice cream bars also contributed to the identification of a sub-clade strongly associated with bars. Some scoop samples analyzed by the enrichment-free enumeration method were produced in November/December 2014 and collected in April 2015.

We need to keep in mind that the method of culture isolation could affect the diversity of food and environmental isolates that were available for analysis, since certain enrichment schemes could preferentially enrich certain genotypes of *L*. *monocytogenes* [42, 43]. The genotypes favored by selective enrichment may be different from those favored during human gastrointestinal passage [44]. In the present study, Cluster I isolates obtained from enrichment-free direct plating enumeration and those obtained from enrichment-based isolation did not form distinct WGS clades, suggesting no bias introduced by selective enrichment, however, we cannot exclude the possibility that the selective agars used for direct plating, Rapid’ *L*. *mono* and Agar *Listeria* Ottaviani and Agosti [5, 24, 25], could introduce bias, although preferential growth of different genotypes on *Listeria* selective agars has not been reported. Isolates representing multiple production lots and environmental locations were all related to the clinical isolates, thus, we did not have strong evidence that there was a significant difference between selective enrichment and human passage in this case.

Previous SNP-based WGS analyses have demonstrated that isolates associated with a common-source outbreak or isolates associated with foods produced in a single facility can differ by up to 5 SNPs [45, 46], 10 SNPs [9], 20 SNPs [47] or 28 SNPs [10]. However, data from those studies are not directly comparable because different WGS analytical tools or SNP calling algorithms were employed. Thus, continuing analysis of isolates associated with listeriosis outbreaks using the same tool(s) is critical to understanding and comparing isolate diversity. Using the CFSAN SNP Pipeline, we have previously showed that isolates associated with a listeriosis outbreak linked to stone fruit differed by up to 42 SNPs [38]. Isolates in WGS Cluster I or Clade IIb differed by up to 29 SNPs and we believe each should represent one strain. In Cluster I, Clades Ia and Ib could be separated by PFGE, but different PFGE profiles in Clade Ib (up to 26 SNPs) did not form distinct clades (Fig 1). Different PFGE profiles in Clade IIb did not form distinct clades either (Fig 3). Therefore, there was no clear evidence of mixed strains in each clade. CFSAN SNP Pipeline applied a filter to remove high density SNPs when analyzing relatively close isolates because high density SNPs could be the result of recombination and/or low quality sequencing/mapping, and low quality mapping often occurs in repetitive regions. We found that 3 removed regions in Cluster I analysis were in prophage regions between tRNA and phage integrase, also part of repeat regions. By using Tandem Repeats Finder [48] and/or examination of annotations and sequences, we found 2 removed regions in Cluster II analysis contained tandem repeats. Our study also helped confirming that the ice cream products analyzed in two studies on *L*. *monocytogenes* enumeration in ice cream and growth kinetics in milkshakes were linked to the outbreak. These two studies revealed that the geometric mean levels of *L*. *monocytogenes* in scoops produced between November 2014 and March 2015 ranged from 0.15 to 7.1 MPN/g [5] and milkshakes prepared from these scoops had a lag phase of 9 h and a growth rate of 0.186 log(CFU)/h when held at room temperature [25]. We sequenced over 70 isolates from those studies, all of which were clustered together with the clinical isolates and showed no bias in sample enrichment (data not shown), but we chose to include only 36 in the present study to avoid over-representing isolates from scoop products.

Prophage variations among Cluster I isolates were primarily gain/loss of prophages, which resulted in the 3 *Asc*I-PFGE profiles observed in all the clinical isolates, as well as in all the food and environmental isolates. We investigated prophage regions because prophage could contain significant variations when the rest of the genome had almost no diversity [9]. Cluster I isolates exhibited multiple PFGE profiles, which allowed comparisons among WGS, PFGE and prophage variations. Isolates exhibiting the same PFGE profile always had the same prophage gain/loss profile and those prophages were conserved, containing two or fewer SNPs. Differences in *Asc*I-PFGE profiles were the result of gain/loss of prophages. In contrast, outbreak-associated isolates and PFGE-matched, non-outbreak isolates had significant prophage divergence. This prophage divergence, often as a result of combination, yields regions containing high density SNPs. The number of such SNPs does not necessarily reflect the actual genetic relatedness between isolates; and thus CFSAN SNP Pipeline excluded these regions for WGS analysis. The Cluster I isolates from the ice cream outbreak described here, CC7 isolates from the 2011 U.S. cantaloupe outbreak [11], and isolates from the 2008 Canada deli meat outbreak [10] all exhibited multiple *Asc*I-PFGE profiles caused by the gain/loss of prophages, indicating that PFGE banding patterns, especially those attributed to prophage gain/loss, may not be sufficient to determine whether an isolate is associated with an outbreak. These different PFGE patterns were observed in different clinical isolates from each outbreak, indicating that gain/loss of prophages did not significantly affect the ability of isolates to cause human illnesses in the aforementioned 3 outbreaks.

PHASTER was an updated version of PHAST for prophage prediction, and they were based on similar algorithms [32, 33]. We found that PHAST was slightly more accurate in identifying prophage insertion sites, while PHASTER was slightly more accurate in the identification of prophage start and end positions, although either software was perfect, as illustrated by the *comK* prophage identification in this study. Examination of tRNA prophage insertion was not conclusive to evaluate PHAST/PHASTER results because the insertion was non-disruptive. This should be kept in mind if these prophages will be used for future in-depth analysis. We also compared the prophages in the ice cream isolate CFSAN029793 and those in the CC5 strain, CFSAN023459, from the 2014 stone fruit recall [38]. The *comK* prophage of the ice cream isolate, CFSAN029793, aligned with *comK* prophage of the stone fruit isolate, CFSAN023459, for 83% coverage and 94% sequence identity, indicating significant prophage divergence (over 2,500 SNPs). The CFSAN029793 prophage 4 differed from the CFSAN023459 prophage 1 by 1 SNP (initially identified by PHAST to be 22.9 Kbp, and an updated analysis using PHASTER revealed it to be 10.7 Kbp). The other two prophages of CFSAN029793 and the other two prophages of CFSAN023459 were totally different (≤ 8% of BLAST alignment coverage) [38]. Thus, strains in the same clonal group but from different outbreaks/incidents had significant prophage divergence, possibly due to recombination, and the resulting high density SNPs were removed when performing WGS analysis of these strains. Our study used a complete genome of CFSAN029793 as the reference for Cluster I, while the best available reference for Cluster II was a draft genome. Therefore, we only performed prophage analysis on Cluster I isolates. We did observe the presence of prophage sequences in Cluster II isolates, which spread across more than one contig of the draft genome, and thus PHASTER analysis could not identify the complete prophage(s) (data not shown). For the same reason, we could only determine whether draft genomes of Cluster I isolates contained the prophages identified in CFSAN029793, but not whether draft genomes contained additional prophages that were not present in CFSAN029793.

Mapping quality and subsequent SNP calling accuracy by reference-based WGS methods may be affected by genetic diversity of isolates [22, 31]. Here we explored the CFSAN SNP Pipeline to analyze CC5 strains from different outbreaks/incidents. Phylogenetic analysis based on identified SNPs successfully separated CC5 strains from non-CC5 strains of the same serogroup. Further, the analysis distinguished CC5 strains from different outbreaks/incidents, which PFGE did not accomplish. However, compared to the SNP analysis on only Cluster I isolates using the same reference, less variant sites were in the final SNP matrix because more sites were filtered out by the software when more diverse genomes were mapped to the reference (data not shown). Thus, the analysis essentially targeted a smaller portion of the genome, as a result, the numbers of pairwise SNP differences among isolates determined were less than those obtained when only Cluster I isolates were analyzed (data not shown); however, this reduced resolution still distinguished the ice cream outbreak-associated isolates from non-outbreak isolates that were matched by PFGE. Therefore, when CFSAN SNP Pipeline is performed on a set of genetically diverse isolates, an initial analysis can be used to identify major clusters; and if pairwise SNP differences need to be precisely determined, additional analyses should be performed on individual clusters. CFSAN SNP Pipeline runs in Linux environment, thus, once the software environment is set up, separate analyses on different clusters are straightforward.

## Conclusions

WGS distinguished outbreak-associated isolates from PFGE/MLST matched, epidemiologically unrelated isolates. WGS also clustered together outbreak-associated isolates exhibiting multiple PFGE profiles. Reference-based SNP analysis allowed simultaneous identification of a CC5 and discrimination of different outbreak strains in the same clone. CFSAN SNP Pipeline can be an effective tool for both long-term and short-term epidemiology of *L*. *monocytogenes*.

## Supporting information

S1 Table Isolates analyzed in the present study. (XLSX)

S2 Table SNPs that specifically differentiate Cluster I from the unrelated isolates, genes containing the SNPs and their encoded proteins. (XLSX)

S3 Table SNPs that specifically differentiate Clade IIb from the unrelated isolates, genes containing the SNPs and their encoded proteins. (XLSX)

S1 Fig Maximum likelihood tree of food and environmental isolates from three facilities as well as clinical isolates. The SNP matrix was generated using CFSAN029793 as the reference. The tree uses midpoint rooting.
(TIF)

S2 Fig Maximum likelihood tree of the Cluster I and epidemiologically unrelated isolates that were matched by PFGE. The SNP matrix was generated using CFSAN029793 as the reference. The tree uses midpoint rooting. In this analysis, Cluster I isolates contained 148 polymorphic loci and differed by 0 to 28 (median, 14) SNPs. The brief PFGE profiles of the unrelated isolates are listed following the isolate ID. The brief PFGE profiles of outbreak-associated isolates are listed under the root of Cluster I.
(TIF)

S3 Fig Maximum likelihood tree of the Cluster II and PFGE-matched, epidemiologically unrelated isolates. The SNP matrix was generated using CFSAN030683 as the reference. The tree uses midpoint rooting. In this analysis, Cluster II isolates contained 165 polymorphic loci and Clade IIb isolates differed by 0 to 28 (median, 16 SNPs) SNPs.
(TIF)

S4 Fig Dendrogram of PFGE profiles associated with the clonal complex 5 isolates. This dendrogram was constructed by Unweighted Pair Group Method with Arithmetic Mean (UPGMA) using *Asc*I-PFGE as the primary pattern and *Apa*I-PFGE as the secondary pattern. The cantaloupe outbreak strain is placed inside the ice cream Cluster I. The cheese outbreak strain is placed inside the ice cream Cluster II.
(TIF)
