# Supplementary figures and images for: Assessing the genome level diversity of Listeria monocytogenes from contaminated ice cream and environmental samples linked to a listeriosis outbreak in the United States
Source: PLoS One. 2017 Feb 6;12(2):e0171389. doi: 10.1371/journal.pone.0171389 (PMC5293252; doi:10.1371/journal.pone.0171389)

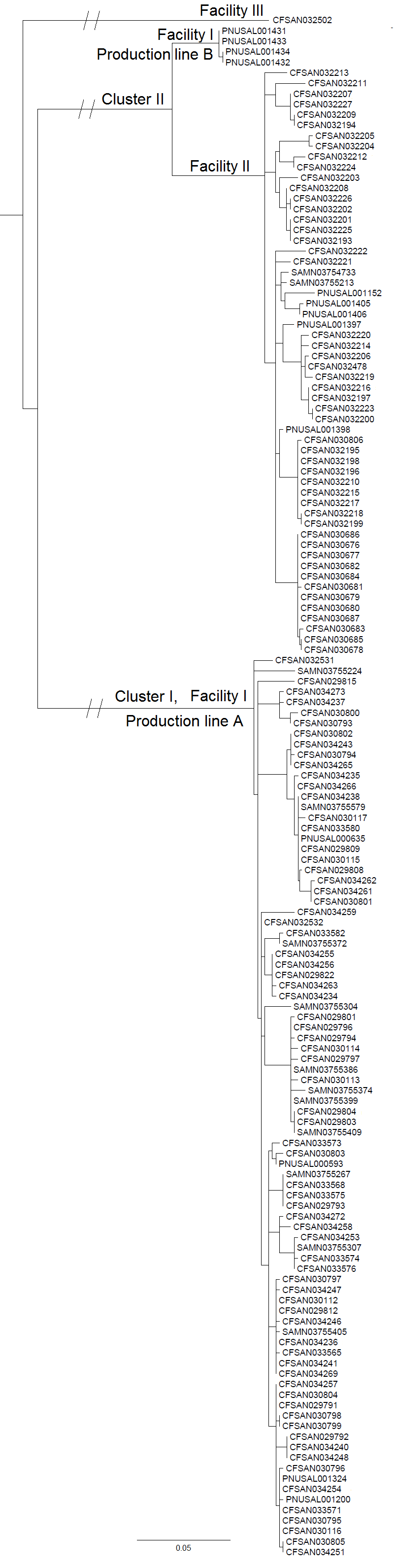

Supplement: S1 Fig — The SNP matrix was generated using CFSAN029793 as the reference. The tree uses midpoint rooting. (TIF) [file pone.0171389.s004.tif]

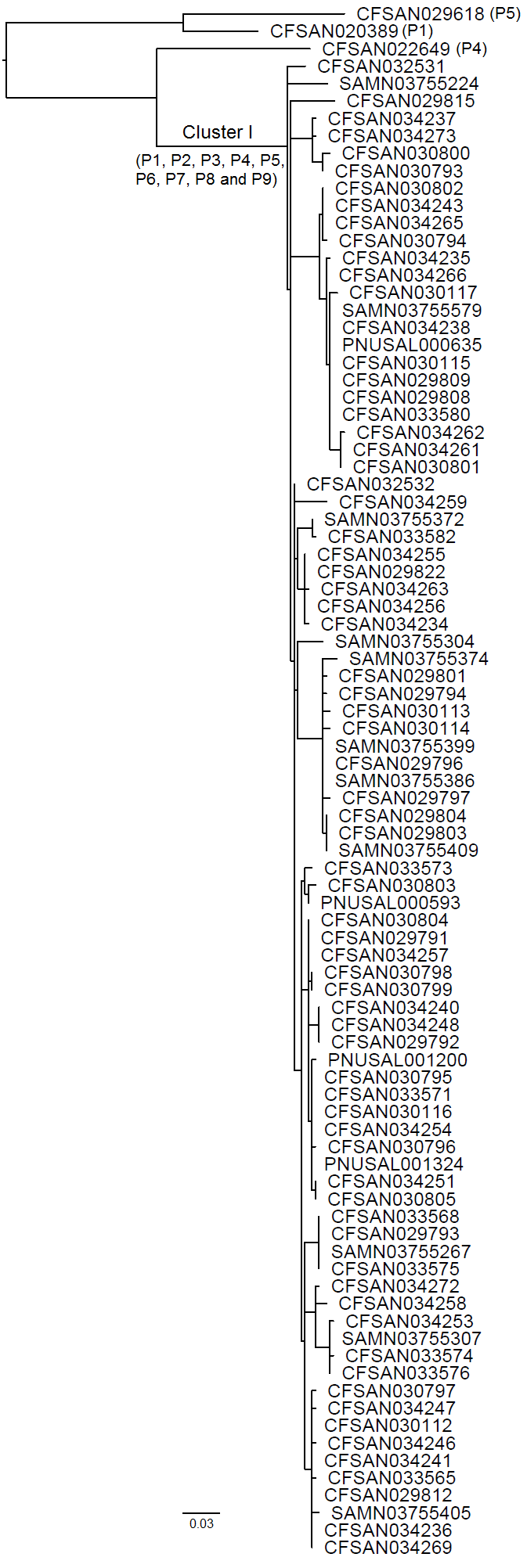

Supplement: S2 Fig — The SNP matrix was generated using CFSAN029793 as the reference. The tree uses midpoint rooting. In this analysis, Cluster I isolates contained 148 polymorphic loci and differed by 0 to 28 (median, 14) SNPs. The brief PFGE profiles of the unrelated isolates are listed following the isolate ID. The brief PFGE profiles of outbreak-associated isolates are listed under the root of Cluster I. (TIF) [file pone.0171389.s005.tif]

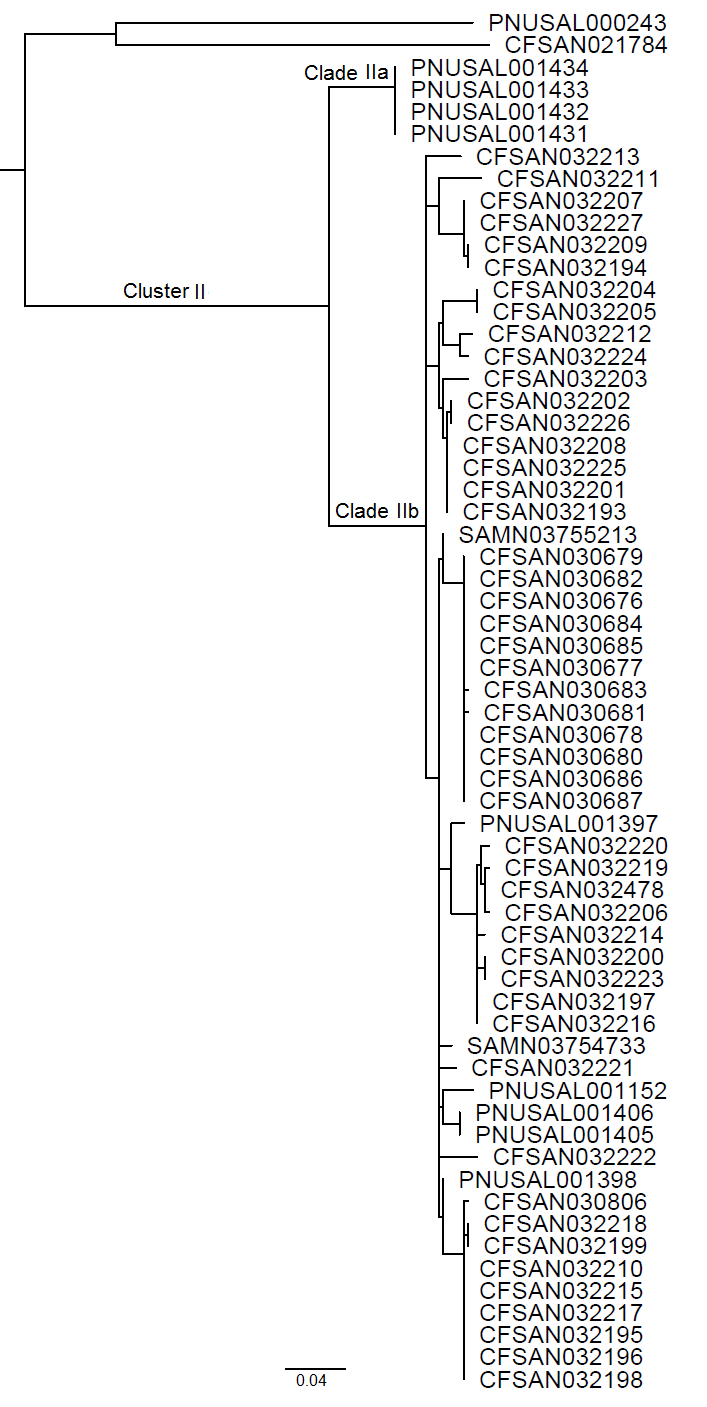

Supplement: S3 Fig — The SNP matrix was generated using CFSAN030683 as the reference. The tree uses midpoint rooting. In this analysis, Cluster II isolates contained 165 polymorphic loci and Clade IIb isolates differed by 0 to 28 (median, 16 SNPs) SNPs. (TIF) [file pone.0171389.s006.tif]

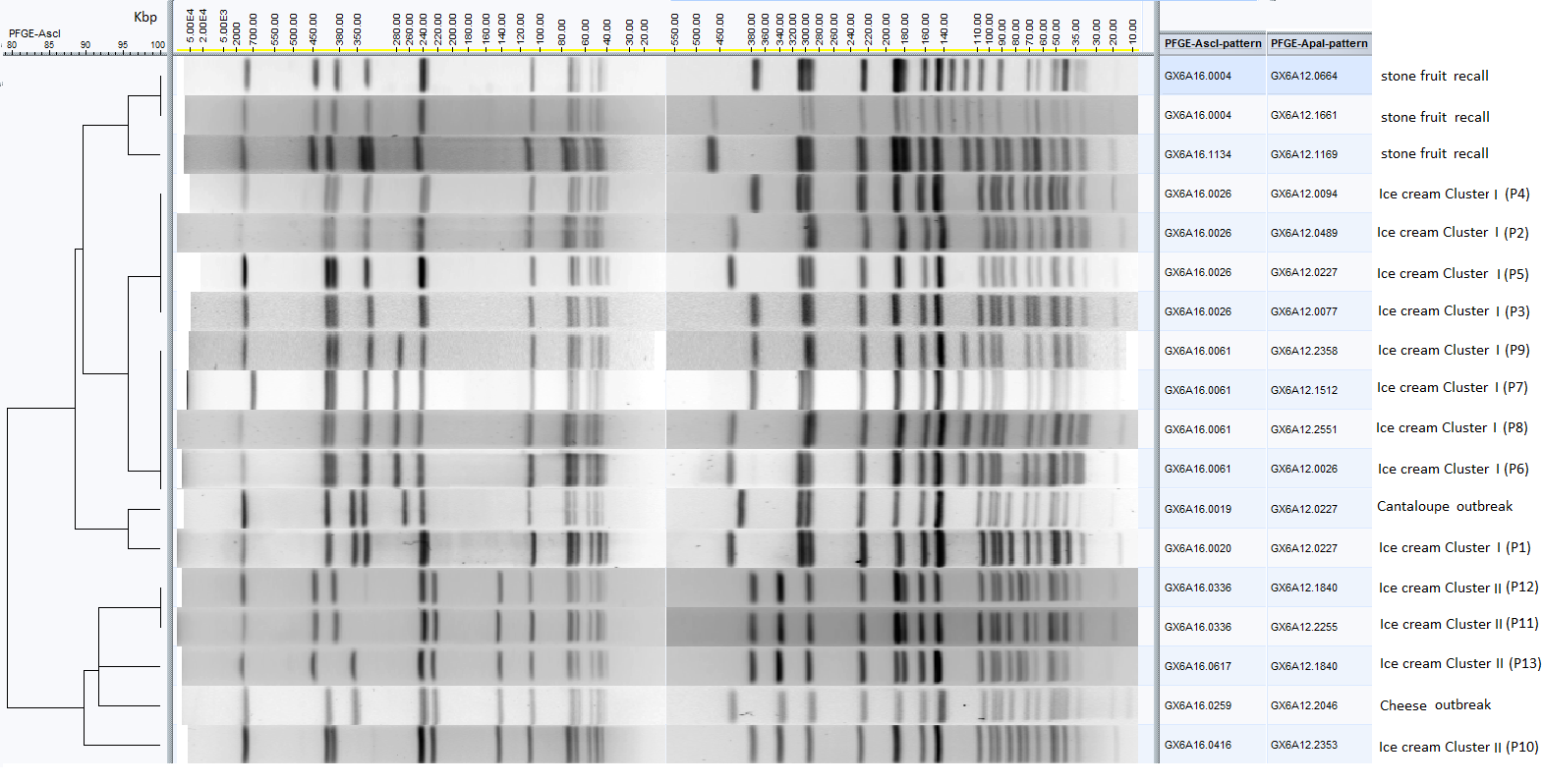

Supplement: S4 Fig — This dendrogram was constructed by Unweighted Pair Group Method with Arithmetic Mean (UPGMA) using AscI-PFGE as the primary pattern and ApaI-PFGE as the secondary pattern. The cantaloupe outbreak strain is placed inside the ice cream Cluster I. The cheese outbreak strain is placed inside the ice cream Cluster II. (TIF) [file pone.0171389.s007.tif]
